# Supplementary material for: Deletion of wfs1 Impairs Oligodendrocyte Precursor Cells Dorsal Distribution and Myelination Through the wfs1-hmgcs1 Axis in Zebrafish
Source: Biology (Basel). 2026 Aug 16;15(16):1402. doi: 10.3390/biology15161402 (PMC13510151; doi:10.3390/biology15161402)
Supplement: Supplementary file 1 [file biology-15-01402-s001.zip › File S1-Transcriptome sequencing data and differentially expressed genes (DEGs) in wfs1-- zebrafish at 5 dpf/1.GO/wfs1KOvsWT/GSEA/wfs1KO/RNA_EXPORT_FROM_NUCLEUS/RNA_EXPORT_FROM_NUCLEUS(GO_0006405).html]

Details for gene set RNA\_EXPORT\_FROM\_NUCLEUS(GO:0006405)[GSEA]


|  || Dataset | deg.sample |
| Phenotype | sample.cls |
| Upregulated in class | wfs1KO |
| GeneSet | RNA\_EXPORT\_FROM\_NUCLEUS(GO:0006405) |
| Enrichment Score (ES) | 0.36675745 |
| Normalized Enrichment Score (NES) | 1.7998502 |
| Nominal p-value | 0.025974026 |
| FDR q-value | 0.130155 |
| FWER p-Value | 0.671 |
Table: GSEA Results Summary

  

Fig 1: Enrichment plot: RNA\_EXPORT\_FROM\_NUCLEUS(GO:0006405)
  
Profile of the Running ES Score & Positions of GeneSet Members on the Rank Ordered List

  

| PROBE | DESCRIPTION   (from dataset) | GENE SYMBOL | GENE\_TITLE | RANK IN GENE LIST | RANK METRIC SCORE | RUNNING ES | CORE ENRICHMENT || 1 | 266985 | npm1a |  |  | 83 | 4.783 | 0.2146 | Yes |
| 2 | 393973 | rae1 |  |  | 554 | 2.834 | 0.3285 | Yes |
| 3 | 563448 | zgc:193690 |  |  | 1628 | 1.584 | 0.3668 | Yes |
| 4 | 492509 | pcid2 |  |  | 3131 | 1.006 | 0.3652 | No |
| 5 | 768163 | chtopb |  |  | 6462 | 0.540 | 0.2852 | No |
| 6 | 445231 | nup85 |  |  | 10935 | 0.248 | 0.1559 | No |
| 7 | 568298 | pom121 |  |  | 11330 | 0.226 | 0.1538 | No |
| 8 | 570158 | alkbh5 |  |  | 11439 | 0.220 | 0.1604 | No |
| 9 | 192325 | ncbp2 |  |  | 11875 | 0.197 | 0.1557 | No |
| 10 | 393917 | ddx39b |  |  | 12078 | 0.186 | 0.1578 | No |
| 11 | 100007012 | si:dkeyp-97b10.3 |  |  | 12184 | 0.180 | 0.1627 | No |
| 12 | 100529857 | nup214 |  |  | 13495 | 0.115 | 0.1268 | No |
| 13 | 571089 | tpra |  |  | 14107 | 0.087 | 0.1115 | No |
| 14 | 445035 | thoc7 |  |  | 14346 | 0.076 | 0.1075 | No |
| 15 | 321491 | chtopa |  |  | 14836 | 0.054 | 0.0946 | No |
| 16 | 323865 | nxf1 |  |  | 14875 | 0.052 | 0.0958 | No |
| 17 | 386850 | eif6 |  |  | 15361 | 0.030 | 0.0819 | No |
| 18 | 558395 | xpo1a |  |  | 15460 | 0.024 | 0.0799 | No |
| 19 | 558883 | tprb |  |  | 16254 | -0.009 | 0.0554 | No |
| 20 | 324551 | zc3h11a |  |  | 17190 | -0.040 | 0.0279 | No |
| 21 | 563392 | nup62l |  |  | 17218 | -0.042 | 0.0289 | No |
| 22 | 286746 | sdad1 |  |  | 17276 | -0.044 | 0.0291 | No |
| 23 | 562350 | nup107 |  |  | 17374 | -0.049 | 0.0283 | No |
| 24 | 571621 | nup153 |  |  | 17848 | -0.069 | 0.0166 | No |
| 25 | 445302 | thoc3 |  |  | 18025 | -0.077 | 0.0145 | No |
| 26 | 327069 | cpsf6 |  |  | 18096 | -0.081 | 0.0160 | No |
| 27 | 393125 | nup155 |  |  | 18319 | -0.090 | 0.0131 | No |
| 28 | 561138 | xpo1b |  |  | 18716 | -0.109 | 0.0056 | No |
| 29 | 541444 | nmd3 |  |  | 18754 | -0.111 | 0.0095 | No |
| 30 | 445408 | gle1 |  |  | 19522 | -0.144 | -0.0080 | No |
| 31 | 436700 | eny2 |  |  | 19586 | -0.147 | -0.0033 | No |
| 32 | 436587 | ltv1 |  |  | 19725 | -0.154 | -0.0007 | No |
| 33 | 30572 | ran |  |  | 20453 | -0.185 | -0.0151 | No |
| 34 | 553507 | npm1b |  |  | 20722 | -0.200 | -0.0145 | No |
| 35 | 436588 | polr2d |  |  | 21291 | -0.230 | -0.0219 | No |
| 36 | 325064 | thoc5 |  |  | 21379 | -0.235 | -0.0139 | No |
| 37 | 100536140 | poldip3 |  |  | 21465 | -0.239 | -0.0057 | No |
| 38 | 436589 | mcm3ap |  |  | 21641 | -0.248 | 0.0000 | No |
| 39 | 559225 | nup188 |  |  | 21691 | -0.251 | 0.0099 | No |
| 40 | 337399 | rrs1 |  |  | 21833 | -0.258 | 0.0172 | No |
| 41 | 560127 | alyref |  |  | 22014 | -0.268 | 0.0237 | No |
| 42 | 373077 | thoc1 |  |  | 23005 | -0.328 | 0.0075 | No |
| 43 | 406324 | abce1 |  |  | 23174 | -0.339 | 0.0176 | No |
| 44 | 368417 | thoc2 |  |  | 23234 | -0.343 | 0.0313 | No |
| 45 | 406852 | nup133 |  |  | 23405 | -0.355 | 0.0421 | No |
| 46 | 558020 | xpot |  |  | 23442 | -0.357 | 0.0572 | No |
| 47 | 445490 | phax |  |  | 23901 | -0.386 | 0.0603 | No |
| 48 | 192339 | ddx19 |  |  | 24093 | -0.399 | 0.0724 | No |
| 49 | 751719 | zgc:153681 |  |  | 24257 | -0.411 | 0.0859 | No |
| 50 | 322269 | shfm1 |  |  | 24451 | -0.417 | 0.0988 | No |
| 51 | 554106 | sarnp |  |  | 24867 | -0.441 | 0.1058 | No |
| 52 | 325550 | ddx39aa |  |  | 25415 | -0.476 | 0.1102 | No |
| 53 | 321744 | nup88 |  |  | 26614 | -0.589 | 0.0993 | No |
| 54 | 323464 | lsg1 |  |  | 27726 | -0.705 | 0.0965 | No |
| 55 | 30172 | nup93 |  |  | 28090 | -0.755 | 0.1194 | No |
Table: GSEA details
[plain text format]

  

Fig 2: RNA\_EXPORT\_FROM\_NUCLEUS(GO:0006405)
  
Blue-Pink O' Gram in the Space of the Analyzed GeneSet

  

Fig 3: RNA\_EXPORT\_FROM\_NUCLEUS(GO:0006405): Random ES distribution
  
Gene set null distribution of ES for
 **RNA\_EXPORT\_FROM\_NUCLEUS(GO:0006405)**
